# Associations of Dietary Inflammatory and Antioxidant Indices with Mental Health Indicators Among University Students: A Cross-Sectional Study

**DOI:** 10.3390/nu17152442

**Published:** 2025-07-26

**Authors:** Merve Esra Çıtar Dazıroğlu, Saniye Bilici, Perim Fatma Türker

**Affiliations:** 1 Department of Nutrition and Dietetics, Faculty of Health Sciences, Gazi University, 06560 Ankara, Türkiye; esracitar@gazi.edu.tr (M.E.Ç.D.); sgbilici@gazi.edu.tr (S.B.); 2 Department of Nutrition and Dietetics, Faculty of Health Sciences, Acıbadem Mehmet Ali Aydınlar University, 34638 İstanbul, Türkiye

**Keywords:** mental health, university student, diet, inflammation, antioxidant

## Abstract

**Background/Objectives:** Protecting students’ mental health during university is essential for their future quality of life. Therefore, diet should be emphasized as a complementary and preventive strategy in supporting and maintaining mental health. This study aimed to examine the association between dietary inflammatory and antioxidant indices and mental health indicators (depression, anxiety, stress, and well-being) in university students. **Methods:** This cross-sectional study included 907 university students. We collected dietary data using a 24 h recall. Based on this data, we used 33 food parameters to calculate the Dietary Inflammatory Index (DII) and 6 antioxidant nutrients to calculate the Dietary Antioxidant Index (DAI). We evaluated mental health using the Depression, Anxiety, and Stress Scales-21 (DASS-21), and assessed well-being using the World Health Organization Five Well-Being Index (WHO-5). **Results:** Overall, 62.4% of participants reported symptoms of depression, 56.2% anxiety, and 40.7% stress. Anxiety and stress levels were significantly higher among females compared to males (*p* < 0.001 and *p* = 0.005, respectively). In fully adjusted models, depression scores were significantly higher in the highest DII tertile compared to the lowest (B = 1.74; 95% CI: 0.24–3.26), while well-being was lower (B = −0.82; 95% CI: −1.65 to −0.00). For DAI, participants in tertile 2 had significantly lower anxiety (B = −1.38; 95% CI: −2.63 to −0.14), depression (B = −1.69; 95% CI: −3.19 to −0.19), and stress (B = −1.70; 95% CI: −3.22 to −0.18) scores compared to tertile 1. No significant association was found between DAI and well-being. **Conclusions:** In this study, university students’ pro-inflammatory diets were associated with poorer mental health profiles. Enhancing the diet’s anti-inflammatory potential may be a promising strategy to support mental health in this population.

## 1. Introduction

Mental health refers to a state of well-being in which an individual realizes their abilities, copes with the everyday stresses of life, works productively, and contributes to the society in which they live [1]. Problems related to mental health, which have a very important place for everyone and everywhere, are unfortunately common [2]. Although mental disorders include a wide range of problems with different symptoms, they are often characterized by a combination of abnormal feelings, thoughts, behaviors, and relationships with others [1]. The latest data from the World Health Organization (WHO) has shown that 970 million people around the world continue to live with mental health problems. The majority of them are anxious, 31.0%, and depressed, 28.9% [2].

University students often struggle with numerous challenges, including increased academic pressures, social adjustments, and economic difficulties. They also become vulnerable to mental health concerns, including anxiety, depression, and stress, due to the stressors associated with navigating a new environment, balancing academic responsibilities, and adapting to greater independence, which can impact their academic performance, personal well-being, and overall quality of life [3]. Also, factors like food insecurity, housing instability, perfectionism, and lack of knowledge of how to shop and cook healthily can further impact their well-being [4].

Previous studies have indicated that biological pathways may affect the relationships between diet quality and mental health, such as the dysregulated hypothalamic–pituitary–adrenal (HPA) axis and inflammation [5].

For mental health, omega-3 fatty acids, phospholipids, cholesterol, niacin, folate, vitamin B_6_, and vitamin B_12_ reflect nutrients that may be beneficial, while saturated fat and simple sugar are considered detrimental to cognitive function [6]. Also, inflammatory dietary patterns may be associated with mental well-being. The brain is particularly vulnerable to oxidative stress as it is rich in lipids in the neuronal membrane and is metabolically active [7,8].

In a systematic review and meta-analysis, it is mentioned that a one-unit increase in the dietary inflammatory index (DII) is associated with a 6% increased risk for symptoms of depression, and an increase in the inflammatory burden of diet was found to be significantly associated with various mental health outcomes [9]. Studies conducted among adolescents and adults suggest that a higher Dietary Antioxidant Index (DAI) may be related to better mental health outcomes. Antioxidants support mental health by reducing oxidative stress, a key contributor to depression, highlighting the potential role of an antioxidant- and anti-inflammatory-rich diet in improving psychological well-being [10]. During the transition to university, students may change their eating habits, such as increasing fast food consumption, relying more on take-out meals, and having a reduced intake of fresh food, which can lead to poor diet quality [11].

Studies investigating the associations between diet quality and mental health have previously focused on the general population, but studies involving the student population in Türkiye are scarce. Also, various studies conducted to date have revealed that the inflammatory load of the diet is associated with multiple mental health problems. However, well-being is less of an issue and addressing this in this study is very important. Moreover, the literature on the effects of DII and dietary antioxidant capacity on mental health and well-being is still limited. Therefore, this study aims to evaluate the impact of the DII and DAI on depression, anxiety, stress, and well-being among university students.

## 2. Materials and Methods

### 2.1. Study Design and Participants

This cross-sectional study was conducted with 907 volunteer university students (268 males, 639 females) in Ankara, the capital city of Türkiye, using convenience and snowball sampling methods. The university students agreed to participate in the study with written informed consent in accordance with the Declaration of Helsinki.

We used a demographic questionnaire, a 24 h dietary recall, the Depression, Anxiety, and Stress Scale (DASS-21), the WHO-5 Well-Being Index, and anthropometric measurements (body weight and height) as data collection tools. Body mass index (BMI) was calculated by dividing weight in kilograms by the square of height in meters (kg/m^2^) [12]. The research data were collected by the students using the face-to-face interview technique.

We excluded individuals with chronic diseases, diagnosed psychological disorders, those using antidepressants, or those following a special diet or eating pattern from the study.

### 2.2. Depression, Anxiety, and Stress Scales

The Depression, Anxiety, and Stress Scales (DASS-21), developed by Lovibond and Lovibond to measure negative emotional states related to depression, anxiety, and stress [13,14], has been adapted into Turkish and validated by Sarıçam (2018) [15]. The scale in question consists of three subscales, namely depression, anxiety, and stress, and includes a total of 21 items, with 7 items in each category. The scale is a 4-point Likert-type scale from 0 to 3 for scoring the items, and it ranges from 0 to 21 for each subscale. Since the DASS-21 is the short version of the original 42-item DASS, the final score for each subscale is multiplied by two (*2). Based on these scores, depression, anxiety, and stress levels are categorized as normal, mild, moderate, severe, or extremely severe [13,14].

### 2.3. World Health Organization (WHO) (Five) Well-Being Index

Developed by the WHO, the WHO (Five) Well-Being Index is a short scale that evaluates emotional well-being in healthy and diseased individuals [16,17]. The Turkish validity and reliability study of the index was conducted by Eser et al. [18]. The scale includes 5 positive statements about the emotions of the participant in the last 2 weeks. Each item on the scale is between 0 and 5 points and is evaluated with a 6-point Likert type scale. A score of 0 on the scale indicates no positive emotions in the last two weeks, and a score of 5 indicates continuous positive emotions. On the scale, where the raw score ranges from 0 to 25 points, the raw score is multiplied by 4 to obtain a percentage score between 0 and 100. The higher the score from the index, the higher the well-being [16,17].

### 2.4. Assessment of Dietary Intake

We used the 24 h dietary recall method to evaluate the participants’ food intake. For this, we asked the participants to record all the food and beverages they consumed the day before. To assess participants’ energy and nutrient intake, we calculated the average daily values using the Nutrition Information System (BeBis) 8.2 program [19].

### 2.5. Assessment of Dietary Inflammatory Index (DII)

We used dietary data from the 24 h recall to calculate participants’ DII scores. DII was first created by Cavicchia et al. in 2009 to measure the overall effect of diet on inflammatory potential [20], and was later developed by Shivappa et al. in 2014 [21]. In this study, we calculated the DII as described in the work of Shivappa et al. [21]. For the calculation, we subtracted the global average daily consumption amounts from the participants’ average daily intake of foods and nutrients, and divided the result by the standard deviation. We converted these to percentiles to reduce the right skewness of the obtained z-scores. Then, we multiplied the percentile values by 2 and subtracted 1 from the result. We multiplied the centered percentile values obtained after this procedure by the customized Inflammatory Effect Score value of that food and nutrient. This way, we calculated the DII value of each food and nutrient. As a result of the sum of these values, the total DII scores from the foods in the diet of the individuals were calculated [21]. Since there are no data on some DII components (flavonoids and spices) these were not included in the calculation of DII in this study, and the calculation was based on a total of 33 nutrients and elements. A resulting negative DII score was associated with an anti-inflammatory effect, while a positive score was associated with a pro-inflammatory effect.

### 2.6. Assessment of Dietary Antioxidant Index (DAI)

We calculated the DAI using the method described by Wright et al. [22] to assess the overall antioxidant capacity of a diet. We determined the intake of six dietary antioxidants (vitamins A, C, and E, magnesium, selenium, and zinc) for all participants. We normalized each micronutrient intake by subtracting the mean intake and dividing by the standard deviation (SD). We summed the resulting standardized values for the six components to obtain the overall DAI score. The detailed calculation formula was as follows.

$$\mathrm{DAI}=\sum_{i=1}^{n=6}\frac{\mathrm{Individual}\;\mathrm{Intake}\;-\mathrm{Mean}}{\mathrm{SD}}$$

### 2.7. Data Analysis

The data collected via the questionnaire were analyzed and interpreted using a statistical software package. The Kolmogorov–Smirnov test was applied to assess the normality of the data distribution. We presented descriptive statistics as frequency (percentage) for categorical variables and mean ± standard deviation (SD) for continuous variables. We used Pearson’s Chi-square test to examine the association between categorical variables. We performed Kruskal–Wallis/Mann–Whitney U analysis to determine the significance of the difference between the means of the groups. We conducted multivariable linear regression analyses to examine the independent effects of the DII and DAI on depression, anxiety, stress, and mental well-being. DII and DAI were included in each model as independent variables, with sex, age, and BMI added as covariates to control for potential confounding. Considering that the study sample predominantly consisted of female participants and individuals with a normal BMI, and that both variables are known potential confounders in the association between diet and mental health, adjusting for them contributed to improving the model accuracy. We examined the relationship between categorical variables using Chi-square analysis. We evaluated results at a 95% confidence interval and considered them statistically significant at a *p* < 0.05 level.

### 2.8. Ethical Considerations

The study protocol was approved by the Ethical Committee of Gazi University (Number: E-77082166-604.01.02-609239, Date: 10 March 2023).

## 3. Results

Table 1 compares sociodemographic characteristics, mental health scores, and dietary indices by gender. Females had significantly lower BMI, mental well-being, and dietary antioxidant index scores, but higher DII scores than males (*p* < 0.05). A substantial proportion of participants experienced depression (62.4%), anxiety (56.2%), and stress (40.7%), with moderate levels appearing to be the most prevalent for each condition. In addition, the prevalence of anxiety and stress was significantly higher among females compared to males (*p* < 0.05).

**Figure 1:**
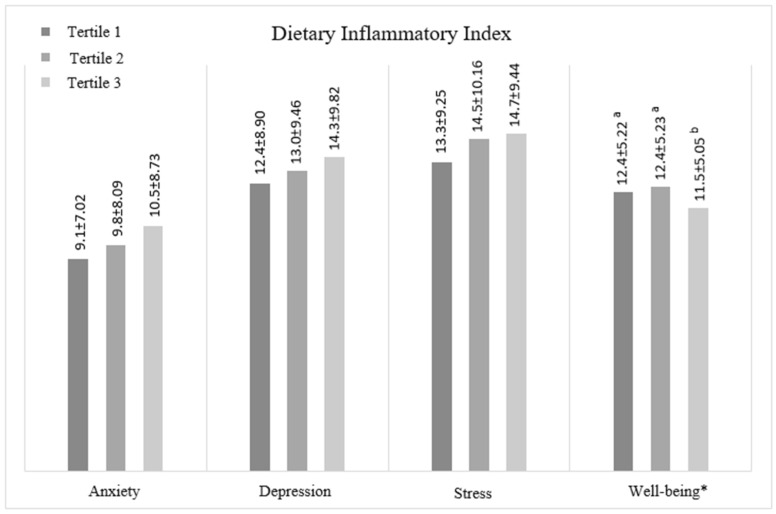
Mean scores of participants’ mental health and well-being traits according to DII. Significant differences (*p* < 0.05) are indicated with an asterisk (*) on the figure. ^a,b^ represent the statistically significant differences among the groups.

Figure 1 shows the mean scores of depression, anxiety, stress, and well-being according to the tertiles of the participants’ DII scores. Accordingly, the mean well-being score in tertile 3 was significantly lower than in both tertiles 1 and 2.

**Figure 2:**
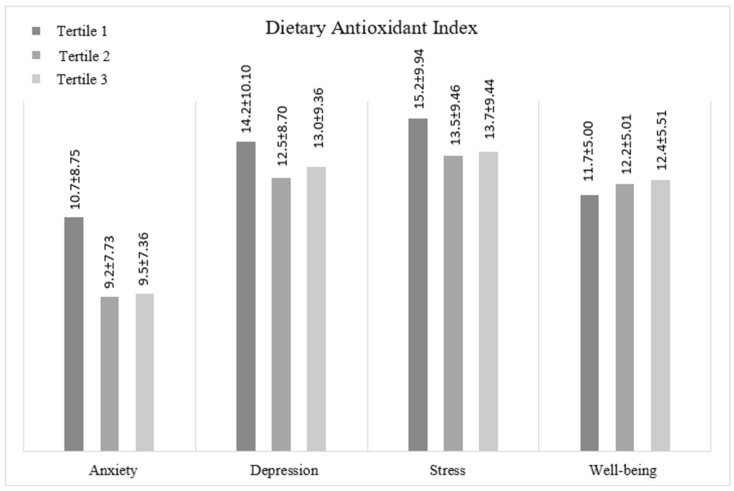
Mean scores of participants’ mental health and well-being traits according to DAI.

The mean scores of depression, anxiety, stress, and well-being according to the tertiles of the participants’ DAI scores are given in Figure 2. Accordingly, we observed no statistically significant difference between the tertiles’ mean values for any mental health indicators.

We conducted multivariable regression analyses based on DII tertiles to examine the associations between DII and depression, anxiety, stress, and well-being (Table 2). According to the initial unadjusted analysis (Model 1), the group with the most pro-inflammatory diet (tertile 3) had significantly higher anxiety (B = 1.33; 95% CI: 0.06–2.61) and depression (B = 1.90; 95% CI: 0.40–3.40) scores compared to the reference group (tertile 1). In addition, well-being scores were significantly lower in this group (B = −0.98; 95% CI: −1.81 to −0.16). After adjusting for sex (Model 2), depression scores remained significantly higher in the tertile 3 group (B = 1.78; 95% CI: 0.27–3.29), and well-being scores remained significantly lower (B = −0.84; 95% CI: −1.67 to −0.01). In Model 3, which was adjusted for both sex and BMI, depression (B = 1.74; 95% CI: 0.24–3.26) and well-being (B= −0.82; 95% CI: −1.65–0.00) scores in the tertile 3 group remained significantly higher.

According to Table 3, which examines the associations between DAI tertiles and depression, anxiety, stress, and well-being, in Model 2 adjusted for sex, anxiety (B: −1.34; 95% CI: −2.59 to −0.09), depression (B: −1.70; 95% CI: −3.21 to −1.00), and stress (B: −1.63; 95% CI: −3.16 to −0.10) scores were significantly lower in the tertile 2 group compared to the tertile 1 group. In Model 3, additionally adjusted for age and BMI, these associations remained significant, and the tertile 2 group had significantly lower anxiety (B: −1.38; 95% CI: −2.63 to −0.14), depression (B: −1.69; 95% CI: −3.19 to −0.19), and stress (B: −1.70; 95% CI: −3.22 to −0.18) scores compared to the tertile 1 group.

## 4. Discussion

In addition to coping with academic pressure, university can be stressful for some students for various reasons, including separation from family and the addition of individualization or job responsibilities. In this context, many university students experience the first onset of mental health problems or the exacerbation of their symptoms during this period [23].

Anxiety and depression are the most common mental health issues among university students, but also problems such as eating disorders, attention deficit/hyperactivity disorder, or schizophrenia can be encountered [23]. A study has shown that 11.94% of university students have anxiety and 7.04% have major depressive disorder [24]. In a study conducted by another group of researchers, it was reported that the prevalence of depression was 17.3% and anxiety was 9.8% [25]. In another study conducted among university students, approximately half of the participants were found to experience depression (48.2%), anxiety (59.8%), and stress (48.2%). Anxiety and stress levels were significantly higher in the female group. When examining severity levels, a greater number of individuals were found in the mild category; however, it is also noteworthy that there were individuals with the highest severity levels, particularly in anxiety, but also in depression and stress [26]. Early detection of these mental health problems, which are common among university students, can reduce attrition and improve educational and psychosocial functionality with effective treatments [27]. In our study, we observed high rates of depression (62.4%), anxiety (56.2%), and stress (40.7%) among participants. Across all three domains, moderate severity levels were the most commonly reported. However, we also observed severe and extremely severe levels in both genders across all three subscales, particularly in the anxiety dimension, indicating that psychological distress was not limited to moderate severity. Furthermore, female participants exhibited significantly higher levels of anxiety and stress than their male counterparts (*p* < 0.05) (Table 1).

DII was developed to determine the inflammatory potential of the diet [21], and this index has been associated with some inflammatory markers such as CRP and IL-6 in various studies [28,29,30,31]. In our study, we found that the majority of participants (approximately 72%) had a BMI within the normal range, suggesting that the influence of obesity-related inflammation may have been limited. Moreover, by including BMI as a covariate in the adjusted regression models, we aimed to ensure that the associations observed between dietary indices (e.g., DII) and mental health outcomes more accurately reflected diet-related effects independent of body weight status. This approach strengthens the interpretability of the findings by minimizing the potential confounding effect of obesity. Low-grade, chronic systemic inflammation is now recognized to be associated with most noncommunicable diseases (NCDs), including obesity, diabetes, cardiovascular diseases, respiratory and musculoskeletal disorders, and cancers, as well as impaired neurodevelopment and adverse mental health outcomes [32]. Relatedly, the focus has been on whether the quality of diet contributes to the psychopathology of widespread mental problems such as depression and anxiety [33]. On the contrary, antioxidants come to the forefront in preventing this and support brain health [34].

In our study, the significant associations between both DII and DAI and depression remained in the fully adjusted models. For DII, depression scores were significantly higher in tertile 3 compared to tertile 1 across all models. In contrast, for DAI, all adjusted models showed that participants in tertile 2 had significantly lower depression scores compared to those in tertile 1 (Table 2 and Table 3). Other investigators have supported similar findings to those in our study, and these studies reported an increased risk of depression among those on the most pro-inflammatory diets [35,36]. For example, women on the most anti-inflammatory diets were found to have an approximately 20% lower risk of developing depression than women on the most pro-inflammatory diet [36]. Salari-Moghaddam et al. [37] found that adherence to a pro-inflammatory diet was positively associated with psychological disorders. Individuals in the highest quintile of the DII score had higher depression compared to those in the lowest quartile (6.56 ± 0.16 vs. 5.48 ± 0.16; *p* < 0.001), anxiety (3.85 ± 0.17 vs. 3.09 ± 0.17; *p* = 0.006), and had psychological distress (2.42 ± 0.13 vs. 1.77 ± 0.13; *p* = 0.001) [37]. Similarly, a pro-inflammatory diet has been associated with a higher risk of various mental health problems in several other studies in different populations [38,39]. Contrastingly, Bergmans et al. [40] found that higher DII scores were also associated with more than twice the likelihood of depression in adults in the US (OR (95% CI) = 1.81 (1.20, 2.71) for the highest vs. lowest quintile, Type III *p*- value = 0.0167), but found no significant results for anxiety [40]. In a study conducted among middle-aged and older American adults, the composite dietary antioxidant index (CDAI) was found to be linearly and inversely associated with depression, and nonlinearly and inversely related to all-cause mortality [41]. Similar findings have been supported in different study populations, highlighting the inverse relationship between depression and CDAI [42,43].

For anxiety, according to the initial unadjusted analysis (Model 1) participants in tertile 3 had significantly higher anxiety scores compared to tertile 1. However, this association lost its statistical significance after adjustments for confounding variables. Regarding DAI, we found that tertile 2 was associated with significantly lower levels of anxiety compared to tertile 1 in all adjusted models (Table 2 and Table 3). Focusing specifically on anxiety, Torabynasab et al. [44] calculated the dietary inflammatory index (EDII) scores of 85 patients with anxiety and healthy individuals (*n* = 85) by taking the frequency of food consumption. As a result, they observed that individuals in the highest category of EDII score were 2.09 times more likely to have an anxiety disorder compared to those in the lower category (OR: 2.09, %95 GA: 1.01, 4.33) [44].

In our study, for DAI all of the adjusted models showed that participants in tertile 2 had significantly lower stress levels than those in tertile 1 (Table 2 and Table 3). In a study conducted with university students living in dormitories in the United Arab Emirates, the association of stress, in addition to anxiety, with EDII scores was highlighted. Logistic regression analysis results showed each point increased anxiety (OR = 1.35; 95% CI: 1.07–1.69; *p* = 0.01) and stress symptoms (OR = 1.41; 95% CI: 1.12–1.77; *p* = 0.003) [45]. Adolescent girls with the highest level of pro-inflammatory diet in Iran were found to have higher stress scores calculated by DASS-21 [46]. Chronic stress contributes to the development of oxidative stress in the brain, while antioxidants play an active role in its prevention and management [47]. However, it is important to emphasize that the relationship is bidirectional. While dietary changes can trigger stress by directly affecting mood, the development of stress can also lead to negative eating behaviors [48]. Stress has been reported to be associated with unhealthy diets in university students [49].

Mental well-being is essential to overall quality of life. When the level of well-being is high, the probability of experiencing mental illness decreases [50]. According to the initial unadjusted analysis (Model 1), participants in the most pro-inflammatory diet group (tertile 3) had significantly lower well-being scores compared to the reference group (tertile 1). This association remained significant after adjustment for sex, with well-being scores still significantly lower in tertile 3 (Table 2). The results of a cross-sectional study showed that while the risk of depression and anxiety is higher in the tertile with the highest EDII score, well-being is lower. Also, this only applies to women [51].

The different results between studies on the relationship of DII and DAI with mental health outcomes may be related to the gender, race, or many other general characteristics of the studied groups. In addition, reasons such as the difference in the scales used to evaluate mental health problems or the non-standardity of the components used for calculating DII or DAI may explain the difference between studies.

This study has some limitations. First, the gender distribution of the sample was unbalanced, with a higher proportion of female participants. Although we adjusted for gender in the regression models to mitigate this effect, caution is warranted when generalizing the findings to a more gender-balanced population. In addition, depression, anxiety, stress, and well-being are closely interconnected, and their impact on each other is undeniable. Therefore, the relationship between these mental health indicators and the nutritional indices examined may reflect a general mental health pattern rather than a domain-specific effect. This possibility should be taken into account when interpreting the results. And finally, we did not assess factors such as smoking status, socioeconomic level, academic performance, physical activity levels, or sleep quality, as these variables were not included in the survey. These unmeasured variables may act as potential confounders and limit the interpretation of the observed associations. Future studies that include these variables and use more representative sampling methods are needed to strengthen the evidence.

## 5. Conclusions

The findings of this study provide additional evidence supporting the relationship between dietary patterns and mental health outcomes among young adults. In this study, higher dietary inflammatory potential, as reflected by increased DII scores, was significantly associated with higher depression levels and lower well-being, even after adjusting for confounding variables. Conversely, moderate dietary antioxidant intake (tertile 2 of DAI) was linked to lower depression, anxiety, and stress scores compared to lower antioxidant intake. These findings suggest that pro-inflammatory and antioxidant dietary patterns may influence young adults’ mental health. University years are essential for mental health, with long-term effects on overall life outcomes. Therefore, supporting students’ nutrition through public catering services in the university environment and designing menus suitable for the Mediterranean diet profile may be beneficial for promoting students’ well-being. Further studies with larger samples are recommended to determine nutritional strategies specific to university students.

## Figures and Tables

**Table 1:** Some general features of participants according to gender.

	Overall (*n* = 907)	Male (*n* = 268)	Female (*n* = 639)	Z	*χ* ^2^	*p*-Value
Age (year), $\bar{X}$ ± SS	21.7 ± 1.63	22.0 ± 1.63	21.6 ± 1.61	−3.826		**0.000**
BMI (kg/m^2^), $\bar{X}$ ± SS	22.6 ± 3.18	24.4 ± 2.98	21.9 ± 2.94	−11.857		**0.002**
BMI category, *n* (%)						
<18.5	61 (6.7%)	4 (1.5%)	57 (8.9%)		100.360	**0.000**
18.5–24.9	651 (71.8%)	152 (56.7%)	499 (78.1%)			
≥25.0	195 (21.5%)	112 (41.8%)	83 (13.0%)			
DASS scores						
Depression, $\bar{X}$ ± SS	13.2 ± 9.42	12.4 ± 8.71	13.6 ± 9.69	−1.169		0.242
Depression status, *n* (%)	566 (62.4%)	167 (62.3%)	399 (62.4%)		28.665	0.122
Normal	341 (37.6%)	101 (37.7%)	240 (37.6%)			
Mild	152 (16.8%)	42 (15.7%)	110 (17.2%)			
Moderate	242 (26.7%)	87 (32.5%)	155 (24.3%)			
Severe	96 (10.6%)	25 (9.3%)	71 (11.1%)			
Extremely severe	76 (8.4%)	13 (4.9%)	63 (9.9%)			
Anxiety, $\bar{X}$ ± SS	9.8 ± 7.99	7.3 ± 6.93	10.9 ± 8.16	−6.571		**0.000**
Anxiety status, *n* (%)	510 (56.2%)	113 (42.2%)	397 (62.1%)		59.916	**0.000**
Normal	397 (43.8%)	155 (57.8%)	242 (37.9%)			
Mild	96 (10.6%)	31 (11.6%)	65 (10.2%)			
Moderate	201 (22.2%)	46 (17.2%)	155 (24.3%)			
Severe	93 (10.3%)	15 (5.6%)	78 (12.2%)			
Extremely severe	120 (13.2%)	21 (7.8%)	99 (15.5%)			
Stress ($\bar{X}$ ± SS)	14.2 ± 9.64	12.4 ± 9.28	14.9 ± 9.70	−3.464		**0.001**
Stress status, *n* (%)	369 (40.7%)	90 (33.6%)	279 (43.7%)		7.950	**0.005**
Normal	538 (59.3%)	178 (66.4%)	360 (56.3%)			
Mild	112 (12.3%)	26 (9.7%)	86 (13.5%)			
Moderate	137 (15.1%)	36 (13.4%)	101 (15.8%)			
Severe	71 (7.8%)	18 (6.7%)	53 (8.3%)			
Extremely severe	49 (5.4%)	10 (3.7%)	39 (6.1%)			
Mental well-being score, $\bar{X}$ ± SS	12.1 ± 5.18	12.9 ± 5.00	11.7 ± 5.22	−3.072		**0.002**
Dietary inflammatory index, $\bar{X}$ ± SS	2.1 ± 1.76	1.8 ± 1.81	2.2 ± 1.72	−3.415		**0.001**
Dietary antioxidant index, $\bar{X}$ ± SS	0.0 ± 3.45	0.7 ± 3.32	−0.3 ± 3.47	−4.268		**0.000**

Statistically significant *p*-values are shown in bold.

**Table 2:** Associations of DII with depression, anxiety, stress, and well-being.

Mental Health Profile	Dietary Inflammatory Index		
	Tertile 1 (*n* = 302)	Tertile 2 (*n* = 303) B (95% CI)	Tertile 3 (*n* = 302) B (95% CI)
Anxiety			
Model 1	Ref	0.73 (−0.54 to 2.00)	1.33 (0.06 to 2.61)
		*p* = 0.261	***p* = 0.041**
Model 2	Ref	0.48 (−0.77 to 1.73)	0.89 (−0.37 to 2.15)
		*p* = 0.449	*p* = 0.164
Model 3	Ref	0.42 (−0.83 to 1.67)	0.88 (−0.38 to 2.13)
		*p* = 0.507	*p* = 0.170
Depression			
Model 1	Ref	0.59 (−0.91 to 2.09)	1.90 (0.40 to 3.40)
		*p* = 0.443	***p* = 0.013**
Model 2	Ref	0.52 (−0.99 to 2.02)	1.78 (0.27 to 3.29)
		*p* = 0.500	***p* = 0.021**
Model 3	Ref	0.48 (−1.02 to 1.98)	1.74 (0.24 to 3.26)
		*p* = 0.533	***p* = 0.023**
Stress			
Model 1	Ref	1.21 (−0.32 to 2.75)	1.43 (−0.11 to 2.97)
		*p* = 0.122	*p* = 0.068
Model 2	Ref	1.05 (−0.49 to 2.58)	1.14 (−0.40 to 2.68)
		*p* = 0.180	*p* = 0.147
Model 3	Ref	0.96 (−0.56 to 2.45)	1.11 (−0.41 to 2.65)
		*p* = 0.216	*p* = 0.152
Well-being			
Model 1	Ref	−0.06 (−0.89 to 0.76)	−0.98 (−1.81 to −0.16)
		*p* = 0.885	***p* = 0.020**
Model 2	Ref	0.02 (−0.81 to 0.84)	−0.84 (−1.67 to −0.01)
		*p* = 0.967	***p* = 0.047**
Model 3	Ref	0.06 (−0.76 to 0.88)	−0.82 (−1.65 to 0.00)
		*p* = 0.891	***p* = 0.050**

Model 1: crude, Model 2: adjusted for sex, Model 3: Additionally adjusted for age and BMI. Statistically significant *p*-values are shown in bold.

**Table 3:** Associations of DAI with depression, anxiety, stress, and well-being. (95% CI).

Mental Health Profile	Dietary Antioxidant Index		
	Tertile 1 (*n* = 303)	Tertile 2 (*n* = 301) B (95% CI)	Tertile 3 (*n* = 303) B (95% CI)
Anxiety			
Model 1	Ref	−1.45 (−2.72 to −0.18)	−1.22 (−2.49 to 0.06)
		***p* = 0.026**	*p* = 0.061
Model 2	Ref	−1.34 (−2.59 to −0.09)	−0.75 (−2.00 to 0.51)
		***p* = 0.035**	*p* = 0.242
Model 3	Ref	−1.38 (−2.63 to −0.14)	−0.69 (−1.95 to 0.56)
		***p* = 0.030**	*p* = 0.277
Depression			
Model 1	Ref	−1.73 (−3.24 to −0.23)	−1.19 (−2.69 to 0.31)
		***p* = 0.024**	*p* = 0.120
Model 2	Ref	−1.70 (−3.21 to −1.00)	−1.04 (−2.55 to 0.47)
		***p* = 0.027**	*p* = 0.175
Model 3	Ref	−1.69 (−3.19 to −0.19)	−0.93 (−2.44 to 0.58)
		***p* = 0.028**	*p* = 0.228
Stress			
Model 1	Ref	−1.71 (−3.24 to −0.17)	−1.53 (−3.06 to 0.01)
		***p* = 0.030**	*p* = 0.051
Model 2	Ref	−1.63 (−3.16 to −0.10)	−1.21 (−2.75 to 0.32)
		***p* = 0.036**	*p* = 0.121
Model 3	Ref	−1.70 (−3.22 to −0.18)	−1.15 (−2.68 to 0.38)
		***p* = 0.029**	*p* = 0.141
Well-being			
Model 1	Ref	0.46 (−0.37 to 1.28)	0.64 (−0.19 to 1.46)
		*p* = 0.279	*p* = 0.131
Model 2	Ref	0.42 (−0.40 to 1.25)	0.49 (−0.34 to 1.31)
		*p* = 0.317	*p* = 0.250
Model 3	Ref	0.43 (−0.39 to 1.26)	0.43 (−0.40 to 1.25)
		*p* = 0.301	*p* = 0.314

Model 1: crude, Model 2: adjusted for sex, Model 3: Additionally adjusted for age and BMI. Statistically significant *p*-values are shown in bold.

## Data Availability

Due to privacy restrictions, the data are available from the corresponding author upon request.
